# Genome-Wide Association Mapping Indicates Quantitative Genetic Control of Spot Blotch Resistance in Bread Wheat and the Favorable Effects of Some Spot Blotch Loci on Grain Yield

**DOI:** 10.3389/fpls.2022.835095

**Published:** 2022-03-03

**Authors:** Philomin Juliana, Xinyao He, Jesse Poland, Sandesh Shrestha, Arun K. Joshi, Julio Huerta-Espino, Velu Govindan, Leonardo Abdiel Crespo-Herrera, Suchismita Mondal, Uttam Kumar, Pradeep K. Bhati, Manish Vishwakarma, Ravi P. Singh, Pawan K. Singh

**Affiliations:** ^1^Borlaug Institute for South Asia (BISA), Ludhiana, India; ^2^International Maize and Wheat Improvement Center (CIMMYT), Texcoco, Mexico; ^3^Biological and Environmental Science and Engineering Division, King Abdullah University of Science and Technology (KAUST), Thuwal, Saudi Arabia; ^4^Department of Plant Pathology, Wheat Genetics Resource Center, Kansas State University, Manhattan, KS, United States; ^5^International Maize and Wheat Improvement Center (CIMMYT), New Delhi, India; ^6^Campo Experimental Valle de Mexico, Instituto Nacional de Investigaciones Forestales, Agricolas y Pecuarias (INIFAP), Chapingo, Mexico

**Keywords:** wheat, spot blotch, GWAS, 2NS translocation, grain yield, *Bipolaris sorokiniana*, CIMMYT

## Abstract

Spot blotch caused by the fungus *Bipolaris sorokiniana* poses a serious threat to bread wheat production in warm and humid wheat-growing regions of the world. Hence, the major objective of this study was to identify consistent genotyping-by-sequencing (GBS) markers associated with spot blotch resistance using genome-wide association mapping on a large set of 6,736 advanced bread wheat breeding lines from the International Maize and Wheat Improvement Center. These lines were phenotyped as seven panels at Agua Fria, Mexico between the 2013–2014 and 2019–2020 crop cycles. We identified 214 significant spot blotch associated GBS markers in all the panels, among which only 96 were significant in more than one panel, indicating a strong environmental effect on the trait and highlights the need for multiple phenotypic evaluations to identify lines with stable spot blotch resistance. The 96 consistent GBS markers were on chromosomes 1A, 1B, 1D, 2A, 3B, 4A, 5B, 5D, 6B, 7A, 7B, and 7D, including markers possibly linked to the *Lr46*, *Sb1*, *Sb2* and *Sb3* genes. We also report the association of the 2NS translocation from *Aegilops ventricosa* with spot blotch resistance in some environments. Moreover, the spot blotch favorable alleles at the 2NS translocation and two markers on chromosome 3BS (3B_2280114 and 3B_5601689) were associated with increased grain yield evaluated at several environments in Mexico and India, implying that selection for favorable alleles at these loci could enable simultaneous improvement for high grain yield and spot blotch resistance. Furthermore, a significant relationship between the percentage of favorable alleles in the lines and their spot blotch response was observed, which taken together with the multiple minor effect loci identified to be associated with spot blotch in this study, indicate quantitative genetic control of resistance. Overall, the results presented here have extended our knowledge on the genetic basis of spot blotch resistance in bread wheat and further efforts to improve genetic resistance to the disease are needed for reducing current and future losses under climate change.

## Introduction

Spot blotch or Helminthosporium leaf blight caused by the hemi-biotrophic fungus *Bipolaris sorokiniana* (Sacc.) Shoemaker [teleomorph: *Cochliobolus sativus* (Ito and Kuribayashi) Drechsler ex Dastur], is a major constraint to bread wheat (*Triticum aestivum*) production in warm and humid wheat-growing regions of the world, threatening the livelihoods of numerous small-holder farmers (Dubin and Van Ginkel, 1991; Duvellier and Gilchrist, 1994; Duveiller et al., 1997; Saari, 1998; Duveiller and Sharma, 2009; Gupta et al., 2018). The disease affects more than 25 million ha of wheat area globally, and is predominant in the intensive rice-wheat cropping systems of eastern India (North-Eastern Plain Zone), Bangladesh, the Terai region of Nepal, South east Asia (Thailand, Philippines, Indonesia, and China), Latin America (Bolivia, warmer regions of Brazil, Paraguay, and northeast Argentina) and Africa (Tanzania and Zambia) (Mehta et al., 1992; Alam et al., 1998; Chang and Wu, 1998; Shrestha et al., 1998; van Ginkel and Rajaram, 1998; Joshi et al., 2002, 2007; Chatrath et al., 2007).

Yield losses due to spot blotch have been substantial and variable depending on the genotypes, sowing time, environmental conditions, soil fertility stresses and soil moisture conditions (Saari, 1998; Sharma and Duveiller, 2004; Duveiller et al., 2005; Duveiller and Sharma, 2009). Estimates of yield losses range from 1 to 20% (Dubin and Van Ginkel, 1991), 2 to 22% in Bangladesh (Siddique et al., 2006), 4 to 43% in the Nepal wheat cultivar Gautam (Sharma and Duveiller, 2006), 43% in Mexico (Villareal et al., 1995), 85% in Zambia (Raemaekers, 1988) etc., but 100% crop loss is also possible under conducive conditions (Mehta, 1985; Saari, 1998). The disease is typically characterized by small dark brown lesions of 1–2 mm length, that extend to form elongated light to dark brown blotches of several centimeters before coalescing and causing leaf necrosis (Mercado Vergnes et al., 2006; Duveiller and Sharma, 2009). This pathogen induced foliar necrosis reduces the photosynthetic area of the leaf and results in premature senescence (Sharma et al., 1997). During favorable conditions, the pathogen can also infect the spikes, resulting in shriveling of the grain, black point of the kernels and deterioration of grain quality (Sharma et al., 1997; Kumar et al., 2002).

Spot blotch management using several agronomic and cultural approaches have been proposed including the use of disease-free seed, optimized sowing time based on the cropping system, timely irrigation, adequate fertilization, crop rotation, removal of infected plant debris, etc., but none of them have been completely effective (Duveiller et al., 2005; Pandey et al., 2005; Sharma and Duveiller, 2006; Sharma P. et al., 2006). While chemical control approaches including seed treatment with fungicides and foliar fungicide application have provided acceptable spot blotch control, their non-affordability by resource poor farmers, the environment and health hazards associated with their use and the possibility of pathogen populations developing resistance to classes of fungicides have limited their usage (Duvellier and Gilchrist, 1994; Duveiller and Sharma, 2009). Hence, the deployment of resistant varieties is the most economical and sustainable spot blotch management strategy, and an integrated approach that combines host-plant resistance as the key component with good agronomic and cultural practices and reasonable chemical control has been recommended (Joshi et al., 2004a; Sharma and Duveiller, 2006; Duveiller and Sharma, 2009).

Genetic resistance to spot blotch and its inheritance has been investigated in several studies that have suggested qualitative and quantitative genetic control of resistance. The first study on the inheritance of seedling resistance to spot blotch in progenies from four inter-varietal crosses indicated the involvement of two dominant complementary genes governing resistance (Srivastava et al., 1971). This was followed by other studies that also suggested the involvement of dominant or partially dominant genes in conditioning spot blotch resistance (Adlakha, 1984; Velázquez Cruz et al., 1994; Sharma and Bhatta, 1999; Neupane et al., 2007). On the contrary, some studies have indicated the involvement of recessive genes in spot blotch resistance (Singh et al., 1998a,^2000^; Bhushan et al., 2002; Ragiba et al., 2004). Further studies by Sharma et al. (1997) and Joshi et al. (2004b) have well established the quantitative and additive genetic inheritance of resistance to spot blotch, respectively.

A critical component in selecting for spot blotch resistance and accelerating breeding efforts involves identifying molecular markers that are closely linked to the resistance loci (Sharma et al., 2007; Duveiller and Sharma, 2009). In a pioneering study on the associations between spot blotch and microsatellite markers, Sharma et al. (2007) reported markers associated with resistance in a population comprising progenies from the cross between spot blotch resistant genotype G162 and susceptible Sonalika. Subsequently, several quantitative trait loci (QTL) mapping studies have identified spot blotch QTL in biparental mapping populations including: Yangmai 6 × Sonalika (Kumar et al., 2009), Ning 8201 × Sonalika and Chirya 3 × Sonalika (Kumar et al., 2010), Bartai × CIANO T79 and Wuya × CIANO T79 (Singh et al., 2018), Wuya × CIANO T79 and Kath × CIANO T79 (Gahtyari et al., 2021) and Bartai × CIANO T79 and Cascabel × CIANO T79 (Roy et al., 2021). In addition, four spot blotch resistance genes have been identified that include: *Sb1* analogous to the durable slow rusting gene *Lr34/Yr18/Sr57/Pm38/Ltn1* (Lillemo et al., 2013), *Sb2* (Kumar et al., 2015), *Sb3* (Lu et al., 2016), and *Sb4* (Zhang et al., 2020).

The application of conventional biparental mapping approaches for the identification of trait linked molecular markers and QTL is limited by the significant population development time involved and the ability to identify only the segregating alleles that are different between the parents for resistance (Brachi et al., 2011; Korte and Farlow, 2013). Hence, an effective alternative to biparental QTL mapping that can utilize available diversity populations (requiring no population development time) and all the historical recombination events that have occurred in a population is genome-wide association mapping, which relies on the linkage disequilibrium (LD) between the causal polymorphisms and markers to identify significant marker-trait associations (Risch and Merikangas, 1996; Remington et al., 2001; Flint-Garcia et al., 2003; Yu and Buckler, 2006). However, very few genome-wide association mapping studies for spot blotch resistance in bread wheat have been reported (Adhikari et al., 2012; Gurung et al., 2014; Ahirwar et al., 2018; Jamil et al., 2018; Juliana et al., 2019; Bainsla et al., 2020; Tomar et al., 2021). Hence, the major objective of this study was to identify consistent (repeatable) markers associated with spot blotch resistance using genome-wide association mapping on a large set of 6,736 advanced breeding lines from the International Maize and Wheat Improvement Center (CIMMYT).

A key strategy that can provide important insights into the allelic composition of lines for trait-associated markers and facilitate informed parental choices by combining desired allelic combinations is allelic fingerprinting, where the favorable and non-favorable alleles at trait-associated markers are fingerprinted (Juliana et al., 2019, 2020a,b). Hence, the second major objective of this study was to fingerprint the 6,736 advanced breeding lines for the favorable and non-favorable alleles at the consistent spot blotch associated markers. We also aimed at using the allelic fingerprinting data to gain a better understanding of the proportion of favorable alleles at the consistent spot blotch associated markers in CIMMYT’s advanced breeding lines. Furthermore, we tested the hypothesis that there is no relationship between the percentage of favorable alleles and spot blotch response of the lines, against the alternate hypothesis that there is a relationship. Finally, we also tested the hypothesis that phenotypic selection for spot blotch from the advanced breeding lines to the Helminthosporium Leaf Blight Screening Nurseries (HLBSNs) in each cycle was effective in increasing the spot blotch favorable allele frequencies in the HLBSNs, against the alternate hypothesis that it was ineffective. The HLBSN comprises about 50 lines each year that are selected from the CIMMYT’s advanced breeding lines for good resistance to spot blotch and other agronomic traits and are distributed to several sites in South Asia and South America, where spot blotch is a major biotic stress (Singh et al., 2018).

## Materials and Methods

### Populations, Spot Blotch Phenotyping, and Statistical Analysis of the Phenotypic Data

We used seven different panels in this study each comprising 1,092 different advanced breeding lines from the CIMMYT bread wheat breeding program’s stage 2 yield trial nurseries, that were evaluated in subsequent crop cycles between 2013–2014 and 2019–2020. The panels were named by the harvesting year of the crop cycle (for example, the panel planted in 2013 and harvested in 2014 is indicated as panel 2014) and include panel 2014, panel 2015, panel 2016, panel 2017, panel 2018, panel 2019, and panel 2020. The stage 2 yield trial nurseries were developed using the selected-bulk breeding scheme (Singh et al., 1998c), in which all the selected plants in early generations are bulked until the head-rows stage, where individual plants are derived from the F_4_, F_5_, or F_6_ generations (depending on the type of cross and the breeding shuttle). Selected lines from the head-rows constitute the stage 1 yield trial nursery (about 9,000 lines), from which lines that have high grain yield, acceptable end-use quality, agronomic type, and phenology, and good resistance to stem and stripe rusts are selected and constitute the stage 2 yield trial nurseries. Since the first evaluation for spot blotch in the breeding cycle is done in the stage 2 yield trial stage, the lines are expected to have good variation for the disease and are considered ideal for mapping.

Spot blotch field response in the stage 2 yield trial nursery lines was evaluated at CIMMYT’s spot blotch screening platform at Agua Fria, Mexico (19° 59′ N, 97° 50′ W), during the 2013–2014 to 2019–2020 crop seasons. The lines were planted during November and harvested in March with four checks namely Chirya 3 (resistant check), Francolin #1 (moderately resistant check), Sonalika (susceptible check), and CIANO T79 (susceptible check). Inoculation was done using a mixture of virulent races that were collected from naturally infected leaf samples in Agua Fria. The double-digit scale (00–99) for rating foliar diseases (Saari and Prescott, 1975; Eyal et al., 1987) was used for scoring spot blotch and four to five disease evaluations between the last week of January and the first week of March were done at weekly intervals. The double-digit scores were then used to calculate the disease severity percentages, from which the area under the disease progress curve (AUDPC) values (Simko and Piepho, 2012) were calculated. The AUDPC values in each panel were expressed as relative AUDPC (rAUDPC) values, relative to the most susceptible line, whose rAUDPC was 100. Days to heading (recorded when about 50% of the plants in a plot had fully emerged spikes) and height (measured in cm from the ground level to the spike tips) were also obtained for all the lines in each of the panels and crop cycles in Agua Fria.

Phenotypic data outliers were detected with the Huber’s robust fit outliers method (Huber and Ronchetti, 2009) using the ‘JMP’ statistical software^1^ and the rAUDPC values that were more than ‘K’ spreads from the center (K was assumed to be 4) were treated as missing. Analysis of spot blotch rAUDPC values in the different panels was done and the mean, standard deviation, median, minimum, maximum, range, and standard error of the mean were obtained. Visualization of the distributions of spot blotch rAUDPC values was done using the ‘R’ package ‘ggplot2’ (Wickham, 2009). Pearson’s correlations between the spot blotch rAUDPC values, days to heading, and height were obtained and *p*-values for the tests of significance of the correlations were also obtained.

### Genotyping

Genome-wide markers were obtained for all the lines in the seven different panels using the genotyping-by-sequencing (GBS) approach (Poland and Rife, 2012; Glaubitz et al., 2014). Single nucleotide polymorphism (SNPs) were called using the Trait Analysis by aSSociation Evolution and Linkage (TASSEL) version 5 and GBS version 2 pipeline (Glaubitz et al., 2014). The SNPs were discovered at a minor allele frequency of 0.01 and Bowtie2 (Langmead and Salzberg, 2012) was used to anchor 6,075,743 unique GBS tags to the first version of the reference sequence assembly of the bread wheat variety Chinese Spring (RefSeq version 1.0) developed by the International Wheat Genome Sequencing Consortium (IWGSC, 2018), with an overall alignment rate of 64% (Juliana et al., 2019). The SNPs were then filtered for those that passed a cut-off of *p* < 0.001 in Fisher’s exact test, had an inbred coefficient value greater than 80%, and a Chi-squared value less than the critical value of 9.2 (given an alpha value of 0.01 and two degrees of freedom). We obtained 78,606 SNPs that passed at least one of these filters and filtered them further for missing data less than 50%, minor allele frequency greater than 5%, and heterozygosity less than 5%. Similarly, the lines with less than 50% missing marker data were filtered and the following number of lines and markers were used for all the subsequent analyses: (i) panel 2014: 904 lines and 7,918 markers (ii) panel 2015: 949 lines and 7,503 markers (iii) panel 2016: 990 lines and 9,695 markers (iv) panel 2017: 1,011 lines and 9,873 markers (v) panel 2018: 962 lines and 8,130 markers (vi) panel 2019: 943 lines and 11,648 markers (vii) panel 2020: 977 lines and 9,507 markers.

### Genome-Wide Association Mapping for Spot Blotch

Genome-wide association mapping for spot blotch was done in all the seven panels using TASSEL version 5 (Bradbury et al., 2007). The mixed linear model (Yu et al., 2006) was fitted, where days to heading, height, and population structure were used as fixed effects, and kinship was used as a random effect. Two principal components accounted for population structure (Price et al., 2006) and kinship was obtained by the centered identity-by-state method (Endelman and Jannink, 2012). For running the mixed linear model, we used the optimum level of compression and the ‘population parameters previously determined’ (Zhang et al., 2010) options in TASSEL.

The *p*-values for the tests of significance of the marker-trait associations, the marker effects, and the percentage of the spot blotch variation explained by each marker were obtained. To ascertain that none of the spot blotch associated significant markers were associated with days to heading and height, we performed genome-wide association mapping for these traits using the mixed linear model and removed any spot blotch-associated marker that was also significantly associated with these traits at a *p*-value threshold of 0.001. We obtained Manhattan plots for all traits showing the −log_10_ *p*-values and chromosomes using the ‘R’ package, ‘CMplot’ (Lilin-yin, 2018). To identify significant markers associated with spot blotch and correct for testing multiple marker-trait associations, we used the Bonferroni correction for multiple testing with an α level of 0.2. The consistent spot blotch associated markers that were significant in more than one panel were obtained and a reference map with those markers aligned to RefSeq version 1.0 was visualized using Phenogram^2^. The LD between the consistent markers was analyzed using TASSEL version 5 and the standardized disequilibrium coefficients (*D*′) (Lewontin, 1964), the correlations between alleles at the two marker loci (*r*^2^), and the *p*-values for the existence of LD using the two-sided Fisher’s Exact test were obtained. Markers with high *r*^2^ values, *D*′ values, and *p*-values for the test of disequilibrium equal to zero were grouped into LD blocks.

### Allelic Fingerprinting of Consistent Spot Blotch Associated Markers

Allelic fingerprinting of the consistent spot blotch associated markers was done for the spot blotch favorable alleles (alleles that had a decreasing effect on the spot blotch rAUDPC values), non-favorable alleles (alleles that had an increasing effect on the spot blotch rAUDPC values), and the heterozygotes in all the 6,736 advanced breeding lines, based on the marker effects that were estimated from the mixed linear model. Since several markers had significant LD amongst themselves, we obtained the consensus allele at an LD block (i.e., the allele that was consistent in at least two markers of the LD block) and all allelic discrepancies among the markers in LD were considered as missing. We then obtained the percentage of lines that had favorable alleles at the spot blotch associated markers or the LD blocks of markers and visualized the allelic fingerprints using the ‘R’ package ‘gplots’ (Warnes et al., 2016).

To understand if there were significant differences between the spot blotch rAUDPC values for the favorable and non-favorable allele at each consistently associated spot blotch marker, we tested the significance of the mean differences in the rAUDPC values for the favorable and non-favorable alleles at each consistent marker and obtained the *p*-values for the two-tailed *t*-tests in each panel. The box plots of the spot blotch rAUDPC values for the alleles at the consistently significant markers were visualized using the ‘R’ package ‘ggplot2’ (Wickham, 2009) for the panels where the mean differences were significant. We performed a two-sided *t*-test to test the hypothesis that there is no relationship between the percentage of favorable alleles at the spot blotch associated markers or the LD block of markers and the spot blotch rAUDPC values of the lines against the alternate hypothesis that there is a relationship. The percentage of favorable alleles in the lines from different panels was plotted against the spot blotch rAUDPC values, using the ‘R’ package ‘ggplot2’ (Wickham, 2009).

To test the hypothesis that phenotypic selection for spot blotch from the advanced breeding lines was effective in increasing the spot blotch favorable allele frequencies in the HLBSNs, against the alternate hypothesis that it was ineffective, we compared the mean rAUDPC values and mean percentage of favorable alleles in the lines from the different panels and the HLBSNs for the following: panel 2014 and 7HLBSN selected from it, panel 2015 and 8HLBSN selected from it, panel 2016 and 9HLBSN selected from it, panel 2017 and 10HLBSN selected from it and panel 2018 and 11HLBSN selected from it.

## Results

### Spot Blotch Phenotypic Data Analysis

**FIGURE 1:**
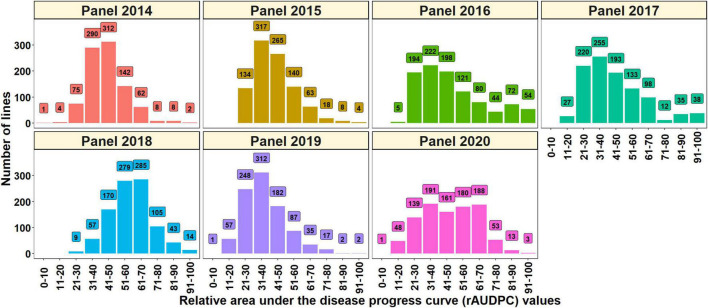
Distribution of the spot blotch relative area under the disease progress curve (rAUDPC) values in panel 2014 (904 lines), panel 2015 (949 lines), panel 2016 (990 lines), panel 2017 (1,011 lines), panel 2018 (962 lines), panel 2019 (943 lines), and panel 2020 (977 lines) during the 2014, 2015, 2016, 2017, 2018, 2019, and 2020 evaluations, respectively.

The distributions of the spot blotch rAUDPC values indicated continuous variation and the mean rAUDPC values ranged between 37.5 ± 13.1 (panel 2019) and 58.9 ± 12.8 (panel 2018) in the different panels (Table 1). Considering the lines with good resistance to spot blotch, we observed that 8.9, 14.1, 20.1, 24.4, 0.9, 32.5, and 19.2% of the lines in panels 2014, 2015, 2016, 2017, 2018, 2019, and 2020, respectively had rAUDPC values less than 30 (Figure 1). We also observed that the rAUDPC values were right skewed in several panels, with 2, 3.2, 17.2, 8.4, 16.8, 2.2, and 7.1% of the lines in panels 2014, 2015, 2016, 2017, 2018, 2019, and 2020, respectively, having high rAUDPC values (>70) and being very susceptible (Figure 1).

**TABLE 1:** Statistical analysis of the relative area under the disease progress curve for spot blotch evaluated in different panels.

	Mean	Standard deviation	Median	Minimum	Maximum	Range	Standard error of the mean
Panel 2014	44.1	11.4	43.1	9.0	100.0	91.0	0.4
Panel 2015	43.2	12.5	41.3	24.7	100.0	75.3	0.4
Panel 2016	49.2	20.5	43.5	15.9	100.0	84.1	0.7
Panel 2017	44.5	18.6	40.6	17.6	100.0	82.4	0.6
Panel 2018	58.9	12.8	58.3	25.8	100.0	74.2	0.4
Panel 2019	37.5	13.1	36.0	9.4	100.0	90.6	0.4
Panel 2020	47.5	16.3	47.0	8.5	100.0	91.5	0.5

Low to high negative correlations were observed between the spot blotch rAUDPC values and days to heading in the different panels that ranged between −0.18 and −0.66 and were highly significant at a *p*-value threshold of 0.001. However, spot blotch rAUDPC values and height had moderately negative correlations of −0.26 and −0.4 in two panels, low negative correlations in three panels (ranged between −0.04 and −0.09), and low positive correlations in the other two panels (0.05 and 0.08). The correlations between spot blotch rAUDPC values and height were significant at a *p*-value threshold of 0.001 in only panel 2015 and panel 2016.

### Marker-Spot Blotch Associations in Different Panels

**FIGURE 2:**
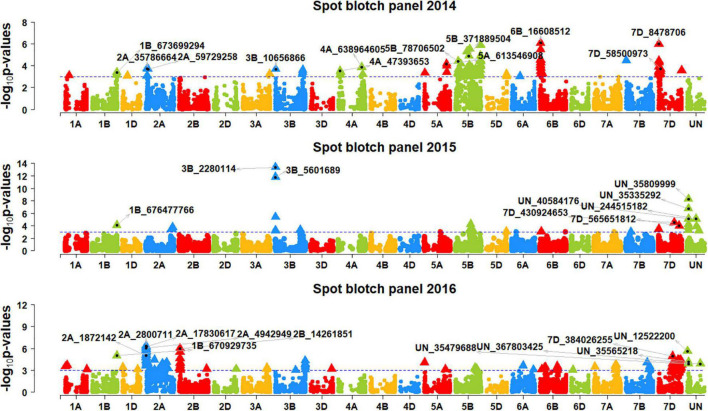
Manhattan plots showing the marker –log_10_ *p*-values and chromosomal positions obtained from genome-wide association mapping for spot blotch in panels 2014, 2015 and 2016. The blue line indicates the *p*-value threshold of 0.001 and selected markers that were either very significantly associated with spot blotch or associated in more than one panel are indicated.

**FIGURE 3:**
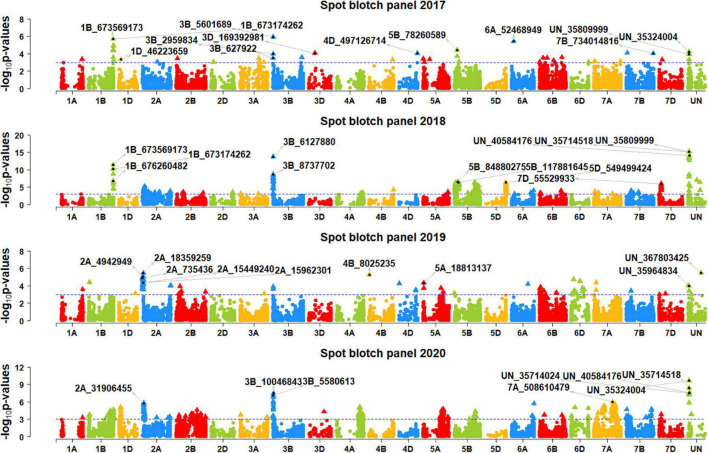
Manhattan plots showing the marker –log_10_ *p*-values and chromosomal positions obtained from genome-wide association mapping for spot blotch in panels 2017, 2018, 2019 and 2020. The blue line indicates the *p*-value threshold of 0.001 and selected markers that were either very significantly associated with spot blotch or associated in more than one panel are indicated.

We performed genome-wide association mapping for spot blotch and identified 892 markers in all the panels that were significantly associated at a *p*-value threshold of 0.001 (Figures 2, 3). This included 98, 30, 170, 47, 261, 87, and 312 significant markers in panels 2014, 2015, 2016, 2017, 2018, 2019, and 2020, respectively. Among them, two markers were significant in four panels, 13 markers were significant in three panels, 81 markers were significant in two panels and 796 markers were significant in one panel only. We then identified markers that were significantly associated with days to heading (Supplementary Figure 1) and height (Supplementary Figure 2) at a *p*-value threshold of 0.001 and removed spot blotch associated markers that were also significantly associated with these traits.

After Bonferroni correction for multiple testing, we obtained 194 markers that were significantly associated with spot blotch, among which only 23 markers were significant in more than one panel, including one marker that was significantly associated in three panels and 22 markers that were significantly associated in two panels. Hence, to avoid losing markers that were not significant after Bonferroni correction, but consistently associated with spot blotch in at least two panels at a *p*-value threshold of 0.001, we have also considered them to be significant. This resulted in 327 significant marker-spot blotch associations in the different panels and 214 unique significant markers (Supplementary Table 1).

In panel 2014, the 29 markers significantly associated with spot blotch were on chromosomes 1B, 2A, 3B, 5B, 5D, 6B, and 7D. Among them, marker 6B_16608512 was the most significant marker that explained 3.1% of the spot blotch variation, followed by markers 7D_8478706, 5B_6873 26022, 5B_680759734, 5B_394982991, 6B_17000615, 5B_68806 3050, and 5B_371889504 that explained 2.5–3% of the variation. In panel 2015, the 15 markers significantly associated with spot blotch were on chromosomes 3B, 5B, 7B, 7D and on unaligned positions. Among them, marker 3B_2280114 was the most significant marker that explained 5.5% of the spot blotch variation, followed by markers 3B_5601689, UN_35809999, UN_35335292, UN_244515182 and UN_40584176 that explained 2.1–4.9% of the variation.

In panel 2016, the 34 markers significantly associated with spot blotch were on chromosomes 1B, 2A, 2B, 5B, 7A, 7D, and on unaligned positions. Among them, marker 2A_2800711 was the most significant marker that explained 2% of the spot blotch variation, followed by markers 2A_17830617, 2A_4942949, 2B_14261851, 2A_18495181, 2A_19902461 and 2A_14418760 that explained 1.9–2% of the variation. In panel 2017, the 18 markers significantly associated with spot blotch were on chromosomes 1B, 1D, 3B, 5B, 6A, and on unaligned positions. Among them, marker 3B_5601689 was the most significant marker that explained 2.1% of the spot blotch variation, followed by markers 1B_673569173, 1B_673174262, 6A_52468949, 1B_677097053, and 1B_673699294 that explained 1.7–2% of the variation.

In panel 2018, the 124 markers significantly associated with spot blotch were on chromosomes 1B, 2A, 3B, 4A, 5B, 5D, 6B, 7B, 7D, and on unaligned positions. Among them, marker UN_35809999 was the most significant marker that explained 7.3% of the spot blotch variation, followed by markers UN_35714518, UN_40584176, 3B_6127880, UN_35335 292, UN_35324004, UN_35565218, UN_34777300, and UN_36 153637 that explained 6.1–7.3% of the variation. In panel 2019, the 31 markers significantly associated with spot blotch were on chromosomes 1A, 2A, 3B, 4B, and on unaligned positions. Among them, marker UN_367803425 was the most significant marker that explained 1.6% of the spot blotch variation, followed by markers 2A_18359259, 4B_8025235, 2A_4942949, 2A_19914469, and 2A_2998843 that explained 1.5–1.6% of the variation. In panel 2020, the 76 markers significantly associated with spot blotch were on chromosomes 1A, 1B, 1D, 2A, 3B, 4A, 5A, 5B, 6A, 6D, 7A, 7B, 7D, and on unaligned positions. Among them, marker UN_35324004 was the most significant marker that explained 4.4% of the spot blotch variation, followed by markers UN_35714518, UN_40584176, 3B_10046843, UN_35714024, UN_34777300, UN_35565218, 3B_5580613, 3B_7237763, and 3B_9107371 that explained 3.1–3.8% of the variation.

### Consistent Marker-Spot Blotch Associations in Different Chromosomes

**FIGURE 4:**
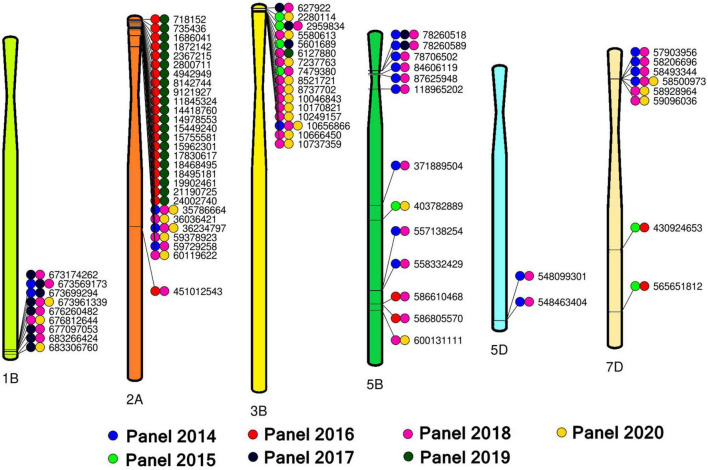
A reference map with 76 spot blotch associated markers significant in two or more panels on chromosomes 1B, 2A, 3B, 5B, 5D, and 7D.

Overall, we have identified 214 markers that were significantly associated with spot blotch in 17 chromosomes, among which 96 markers on 12 chromosomes were significantly associated in more than one panel. This included 28 markers on chromosome 2A, 16 markers on chromosome 3B, 13 markers on chromosome 5B, nine markers on chromosome 1B, eight markers on chromosome 7D, two markers on chromosome 5D and one marker each on chromosomes 1A, 1D, 4A, 6B, 7A, and 7B and 14 unaligned markers. We have also created a reference map (Figure 4) with 76 of the 96 spot blotch associated consistent markers that were significant in more than one panel (excluding the chromosomes that had only one significant marker and the unaligned markers).

On chromosome 1BL, nine markers between 673174262 and 683306760 bps were significantly associated with spot blotch, among which markers 1B_673569173 and 1B_673961339 were significant in three panels. Among these, significant LD was observed among markers 1B_673174262, 1B_673569173, 1B_673699294, and 1B_673961339 (*D*′ values ranged between 0.91 and 0.99), markers 1B_676260482, 1B_676812644 and 1B_677097053 (*D*′ values ranged between 0.98 and 0.99) and markers 1B_683266424 and 1B_683306760 (*D*′ value was 1) (Supplementary Figure 3).

On chromosome 2AS, 21 markers between 718152 and 24002740 bps were significantly associated with spot blotch. Analysis of LD among these markers indicated a single LD block (henceforth this LD block is referred to as 2A_718152-2A_24002740), with significant LD among all the markers (*D*′ values ranged between 0.94 and 1) except marker 2A_9121927 (Supplementary Figure 4). In addition, three other markers on chromosome 2AS (2A_35786664, 2A_36036421 and 2A_362 34797) formed an LD block (*D*′ values ranged between 0.94 and 0.99) among which markers 2A_35786664 and 2A_36234797 were significantly associated in three panels. Furthermore, markers 2A_59378923, 2A_59729258, and 2A_60119622 on chromosome 2AS also formed an LD block (*D*′ values ranged between 0.89 and 1).

On chromosome 3BS, 16 markers were significantly associated with spot blotch in more than one panel, among which markers 3B_2959834 and 3B_10656866 were significantly associated in three panels. Among these 16 markers, we observed that markers 3B_7237763, 3B_8521721, and 3B_8737702 had significant LD (*D*′ values ranged between 0.99 and 1), in addition to markers 3B_10170821, 3B_10249157, 3B_10656866, and 3B_10666450 (*D*′ values ranged between 0.96 and 1) that also formed an LD block (Supplementary Figure 5). However, besides these seven markers, the other nine consistently significant markers on chromosome 3BS did not have significant LD with other markers.

On chromosome 5BS, six markers between 78260518 and 118965202 bps (henceforth referred to as 5B_78260518-5B_118965202) were significantly associated with spot botch in more than one panel and formed an LD block (*D*′ values ranged between 0.97 and 1, Supplementary Figure 6). Among them, markers 5B_78260518 and 5B_78260589 were associated with spot blotch in three panels. On chromosome 5BL, the marker pairs that were significantly associated with spot botch in two panels and formed an LD block included 5B_557138254 and 5B_558332429 (*D*′ = 1) and 5B_586610468 and 5B_586805570 (*D*′ = 1). On chromosome 5DL, markers 5D_548099301 and 5D_548463404 were significantly associated with spot botch in two panels and formed an LD block (*D*′ = 0.98). On chromosome 7DS, markers 7D_57903956, 7D_58206696, 7D_58493344, and 7D_58500973 were significantly associated with spot blotch in more than one panel and formed an LD block (*D*′ values ranged between 0.98 and 1, Supplementary Figure 7), among which marker 7D_58500973 was significantly associated in three panels. We also observed that marker pairs 7D_58928964 and 7D_59096036 (*D*′ = 0.98) on chromosome 7DS and markers 7D_430924653 and 7D_565651812 (*D*′ = 1) on chromosome 7DL were significant in more than one panel and formed LD blocks.

Among the 14 unaligned markers that were associated with spot blotch in more than one panel, markers UN_12522200 and UN_367803425 had high LD with the markers between 718152 and 24002740 bps on chromosome 2AS (excluding marker 2A_9121927) and are probably indicating the same QTL (*D*′ values ranged between 0.96 and 1, Supplementary Figure 8). Among the remaining unaligned markers, UN_35324004, UN_3 4777300, UN_35335292, UN_35565218, UN_35714024, UN_35 714518, UN_35809999, UN_40584176, UN_244515182, and UN _326649097 had significant LD among themselves (Supplementary Figure 8) and with markers on chromosome 3BS. Markers UN_35565218, UN_35714518 and UN_40584176 were in significant LD with marker 3B_2959834 (*D*′ values ranged between 0.91 and 0.93). Markers UN_35335292 and UN_358 09999 were in significant LD with marker 3B_6127880 (*D*′ values ranged between 0.95 and 0.99). Markers UN_34777300, UN_35324004, UN_35714024, UN_244515182, and UN_3266 49097 were in significant LD with marker 3B_8737702 (*D*′ values ranged between 0.86 and 0.98).

### Allelic Fingerprinting of Consistent Spot Blotch Associated Markers

**FIGURE 5:**
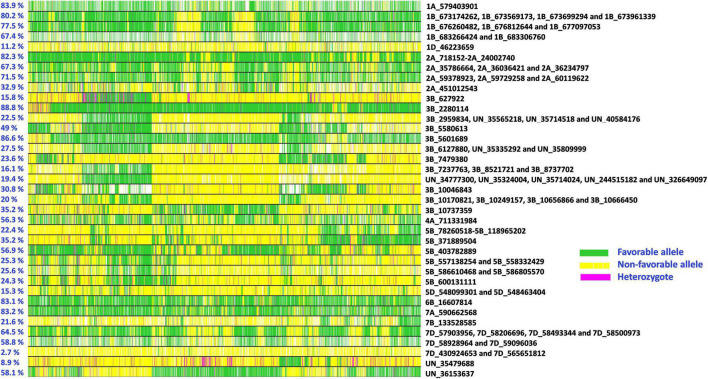
Allelic fingerprinting of spot blotch associated markers. The percentage values on the left indicate the percentage of lines with favorable alleles at the markers. The green color indicates the favorable allele (allele that has a decreasing effect on spot blotch), the yellow color indicates the non-favorable allele (allele that has an increasing effect on spot blotch), the magenta color indicates the heterozygote, and the white color indicates missing data.

Allelic fingerprinting of consistent spot blotch associated markers in the advanced breeding lines (Figure 5 and Supplementary Table 2) indicated that the percentage of favorable alleles at markers 3B_2280114 (88.8%), 3B_5601689 (86.6%), 1A_579403901 (83.9%), 7A_590662568 (83.2%), and 6B_16607814 (83.1%), and the percentage of consensus favorable alleles in the 2A_718152 – 2A_24002740 LD block (82.3%), and the LD block tagged by markers 1B_673174262, 1B_673569173, 1B_673699294, and 1B_673961339 (80.2%) were high. Similarly, the percentage of consensus favorable alleles at LD blocks tagged by markers 1B_676260482, 1B_676812644, and 1B_677097053 (77.5%); 2A_59378923, 2A_59729258, and 2A_60119622 (71.5%); 1B_683266424 and 1B_683306760 (67.4%); 2A_35786664, 2A_36036421, and 2A_36234797 (67.3%) and 7D_57903956, 7D_58206696, 7D_58493344, and 7D_58500973 (64.5%) were greater than 50%. We also observed a range in the percentage favorable alleles of the spot blotch associated markers on chromosome 3BS between 627922 and 10737359 bps (15.8–88.8%). Considering the lines with a high number of spot blotch favorable alleles, we observed that 267 lines had favorable alleles at 67 or more of the 96 markers (Supplementary Table 3).

### Effects of the Favorable and Non-favorable Alleles on the Spot Blotch Relative Area Under the Disease Progress Curve Values

Tests of significance of the mean differences in the spot blotch rAUDPC values between the lines with the favorable alleles and the non-favorable alleles were performed for all the 37 spot blotch associated markers/LD blocks in the different panels. Among the 259 two-tailed *t*-tests of significance, only 131 tests of mean differences were significant at a *p*-value threshold of 0.001 (Supplementary Figure 9). Among them, we observed that the mean rAUDPC values were significantly different in all the seven panels for the favorable and non-favorable alleles at the LD blocks tagged by markers: (i) 5B_557138254 and 5B_558332429, (ii) 5B_586610468 and 5B_586805570, and (iii) 7D_57903956, 7D_58206696, 7D_58493344, and 7D_58500973.

### Relationship Between the Percentage of Favorable Alleles at the Significant Spot Blotch Associated Markers and the Spot Blotch Relative Area Under the Disease Progress Curve Values

**FIGURE 6:**
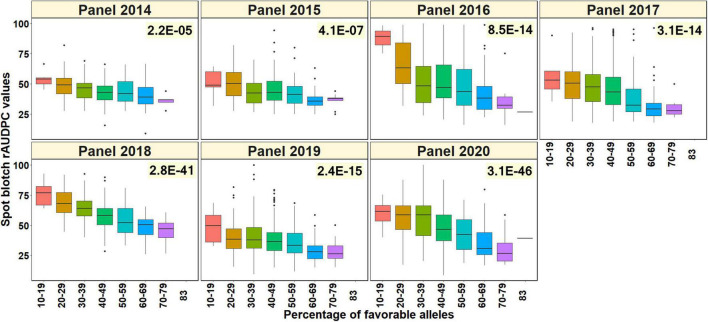
Percentage of favorable alleles (alleles that have a decreasing effect on spot blotch) in the lines from different panels plotted against the spot blotch relative area under the disease progress curve (rAUDPC) values in seven panels. The values on the top right of the panels indicate the two-sided *p*-values for the test that there is no relationship between the percentage of favorable alleles and the spot blotch rAUDPC values of the lines against the alternate hypothesis that there is a relationship.

To test if the percentage of favorable alleles at the spot-blotch associated markers was associated with the spot blotch response of the lines, we obtained a subset of 3,608 lines that had non-missing data in at least 30 of the 37-spot blotch associated markers/LD blocks. This included 240, 285, 508, 521, 499, 766, and 789 lines from panels 2014, 2015, 2016, 2017, 2018, 2019, and 2020, respectively. The two-sided *t*-test *p*-values indicated that in all the panels there was a significant relationship between the percentage of favorable alleles in the lines and their spot blotch rAUDPC values, with the *p*-values ranging between 2.2E-05 in panel 2014 and 3.1E-46 in panel 2020 (Figure 6).

### Relationship Between Alleles at Selected Significant Spot Blotch Associated Markers and Grain Yield

The association of some of the significant spot blotch associated markers in this study (2A_718152 – 2A_24002740, 3B_2280114, and 3B_5601689) with grain yield has been reported in previous studies (Juliana et al., 2019, 2021). Hence, our objective was to understand the relationship between the spot blotch favorable and non-favorable alleles and grain yield in different environments that have been reported in Juliana et al. (2021). So, for the advanced breeding lines in this study, we used the grain yield data obtained from the following testing environments: (i) stage 1 irrigated-bed planting environment where the CIMMYT bread wheat breeding program’s stage 1 yield testing nursery lines were evaluated at the Norman E. Borlaug Experimental Research Station, Ciudad Obregon, Mexico (27°29′N, 109°56′W) on raised beds and received optimum irrigation of about 500 mm of water in total from five irrigations. (ii) Stage 2 irrigated-bed planting and irrigated-flat planting environments where CIMMYT’s stage 2 yield testing nursery lines were evaluated in Obregon on raised beds and flatbeds, respectively, under optimum irrigation. (iii) Stage 2 moderate-drought stress environment where CIMMYT’s stage 2 yield testing nursery lines were evaluated in Obregon on raised beds under moderate-drought stress, with irrigation of about 200 mm of water in total from two irrigations. (iv) Stage 2 severe-drought stress environment where CIMMYT’s stage 2 yield testing nursery lines were evaluated in Obregon under severe-drought stress in the flat planting system, with a total of about 180 mm of water through drip irrigation. (v) South Asia bread wheat genomic prediction yield trial environments where a subset of CIMMYT’s Stage 2 yield trial nursery lines (540 lines) was evaluated in India at the Borlaug Institute for South Asia stations in Jabalpur, Madhya Pradesh (23° 10′ N, 79° 55′ E), Ludhiana, Punjab (30° 54′ N, 75° 51′ E) and Pusa, Bihar (25° 59′ N, 85° 41′ E) in the flat planting system under optimum irrigation. Further description of the crop cycles of evaluation, trial management conditions, and experimental designs in all these environments is available in Juliana et al. (2021).

**FIGURE 7:**
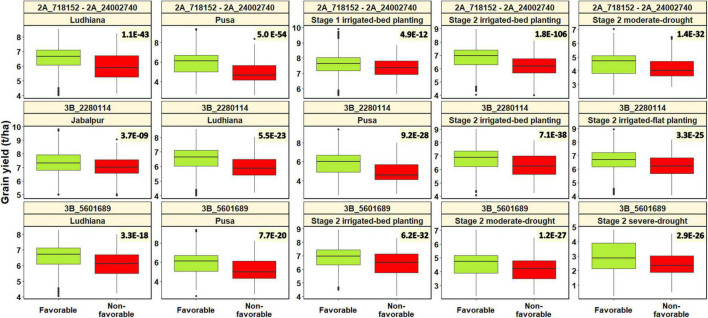
Spot blotch favorable alleles (alleles that have a decreasing effect on spot blotch) and non-favorable alleles (alleles that have an increasing effect on spot blotch) at linkage disequilibrium block 2A_718152 – 2A_24002740 and markers 3B_2280114 and 3B_5601689 plotted against the grain yield in different environments. The values on the top right of the panels indicate the two-sided *t*-test *p*-values for the test that there is no relationship between the spot blotch associated marker alleles and grain yield against the alternate hypothesis that there is a relationship.

For testing the association of the significant spot blotch-associated marker alleles (2A_718152 – 2A_24002740, 3B_2280114, and 3B_5601689) with grain yield, we used combined panels with all the fingerprinted stage 1 yield trial, stage 2 yield trial, and the South Asia bread wheat genomic prediction yield trial lines. The best linear unbiased estimates for grain yield (t/ha) across all the panels and years were obtained as described in Juliana et al. (2021). We also visualized the differences in grain yield (t/ha) between the lines with the spot blotch favorable alleles and the non-favorable alleles at the three genomic regions using the ‘R’ package ‘ggplot2’ (Wickham, 2009), and performed two-sided *t*-tests of the significance of the mean differences in grain yield between the lines with and without the spot blotch favorable alleles. We observed that the spot blotch consensus favorable allele for the markers in the 2A_718152 – 2A_24002740 LD block had a significant effect on grain yield evaluated in Stage 2 irrigated-bed planting environment, Pusa, Ludhiana, Stage 2 moderate-drought stress environment, and Stage 1 irrigated-bed planting environment, with *p*-values ranging between 4.9E-12 and 1.8E-106 (Figure 7). The effects of the 2A_718152 – 2A_24002740 LD block on grain yield were 0.49 t/ha (Pusa), 0.31 t/ha (Stage 2 irrigated-bed planting environment), 0.30 t/ha (Ludhiana), 0.18 t/ha (Stage 2 moderate-drought stress environment), and 0.11 t/ha (Stage 1 irrigated-bed planting environment) in different environments.

Marker 3B_2280114 had a significant effect on grain yield in the Stage 2 irrigated-bed planting environment, Pusa, Stage 2 irrigated-flat planting environment, Ludhiana, and Jabalpur, with *p*-values ranging between 3.7E-09 and 7.1E-38. The spot blotch favorable allele at marker 3B_2280114 had effects of 0.48 t/ha (Pusa), 0.29 t/ha (Ludhiana), 0.25 t/ha (Stage 2 irrigated-bed planting environment), 0.20 t/ha (Stage 2 irrigated-flat planting environment), and 0.17 t/ha (Jabalpur) on grain yield in different environments. Marker 3B_5601689 had a significant effect on grain yield in the Stage 2 irrigated-bed planting environment, Stage 2 moderate-drought environment, Stage 2 severe-drought environment, Pusa and Ludhiana, with *p*-values ranging between 3.3E-18 and 6.2E-32. The spot blotch favorable allele at marker 3B_5601689 had effects of 0.37 t/ha (Pusa), 0.25 t/ha (Stage 2 severe-drought stress environment), 0.23 t/ha (Ludhiana), 0.22 t/ha (Stage 2 irrigated-bed planting environment), and 0.22 t/ha (Stage 2 moderate-drought stress environment) on grain yield in different environments.

### Mean Relative Area Under the Disease Progress Curve Values and Mean Percentage of Favorable Alleles in the Lines From the Different Panels and the Helminthosporium Leaf Blight Screening Nurseries

**FIGURE 8:**
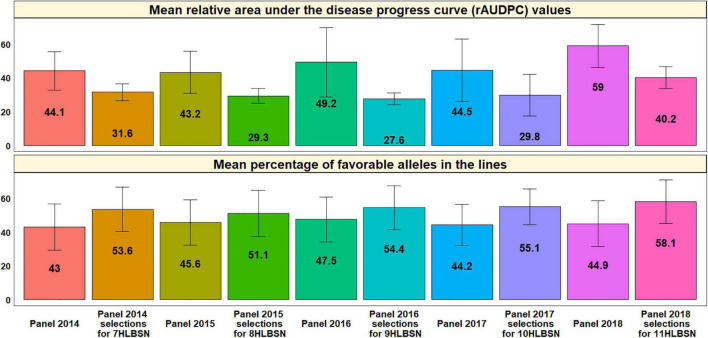
Mean relative area under the disease progress curve (rAUDPC) values and mean percentage of favorable alleles in the lines from the different panels and selections from the panels that constitute the Helminthosporium Leaf Blight Screening Nurseries (HLBSNs).

Analysis of the mean rAUDPC values and percentage of favorable alleles in the lines from the different panels and HLBSNs derived from the different panels indicated that the HLBSNs selected from all the panels had significantly lower mean rAUDPC values (*p*-value = 0.002) and also significantly higher mean percentage of favorable alleles (*p*-value = 0.0001) at a *p*-value threshold of 0.005 (Figure 8). We also observed that the mean rAUDPC values ranged between 43.2 +12.5 and 58.9 +12.8 in the panels, while they ranged between 27.6 +3.5 and 40.2 +6.3 in the HLBSNs. Similarly, the mean percentage of favorable alleles in the panels ranged from 43 +13.6% (panel 2014) to 47.5 +13.2% (panel 2016), while they ranged from 51.1 +13.7% (8HLBSN) to 58.1 +12.8% (11HLBSN) in the different HLBSNs.

## Discussion

In this study, we analyzed the distributions of spot blotch rAUDPC values in advanced breeding lines from CIMMYT and our results showed that on average 17.2% of the lines had low rAUDPC values (<30) and 8.1% of the lines had very high rAUDPC values (>70). None of the lines had rAUDPC values less than 5 and a majority of the lines in the different panels had intermediate rAUDPC values. These results reaffirm previous reports on the lack of immune genotypes for spot blotch (Mehta, 1998; Duveiller et al., 2005; Khan and Chowdhury, 2011) and also indicate that a large number of advanced lines from the CIMMYT bread wheat breeding program possess moderate resistance to spot blotch, due to extensive breeding efforts (Singh et al., 2015, 2016).

Our results also indicated significant low to high negative correlations between the spot blotch rAUDPC values and days to heading in Agua Fria. These results are consistent with previous studies that have reported negative correlations between spot blotch and days to heading, indicating that late genotypes tend to possibly escape the disease (Sharma et al., 1997; Dubin et al., 1998; Sharma R. C. et al., 2006; Singh et al., 2015). However, CIMMYT breeders evade the association of growth stage with spot blotch in selections, by comparing disease responses to both early and late checks. We also observed mostly non-significant and inconsistent positive to negative correlations between the spot blotch rAUDPC values and height as previously observed (Sharma R. C. et al., 2006; Singh et al., 2015). We attribute the changes in magnitude and direction of correlations between spot blotch and height in different panels to the use of diverse parents in developing the lines in each panel (Sharma et al., 1997) and strong selection for height in early generations of the breeding program, which results in a narrow variation for height in the advanced breeding lines. However, the low negative correlations of days to heading with spot blotch and no correlation of height with spot blotch in some panels also indicate the independence of these traits and suggest that selection for early heading, short and spot blotch resistant lines is possible, as also observed in previous studies (Joshi et al., 2002; Sharma R. C. et al., 2006; Singh et al., 2015).

We have successfully used genome-wide association mapping for dissecting the genetic architecture of resistance to spot blotch in CIMMYT’s advanced bread wheat breeding lines and identified 96 consistent GBS markers associated with spot blotch on twelve chromosomes. A key observation in this study is that among the 214 significant markers in all the panels, only 96 were significant in more than one panel and only two markers were significant in four panels. While this could be partly attributed to the variable allele frequencies and marker missing data in the different panels for some markers, it also indicates a strong effect of genotype × environment interactions (Singh et al., 2015; Mahapatra et al., 2020; Chattopadhyay et al., 2021; Roy et al., 2021) in identifying consistent spot blotch associated markers, and highlights the need for multiple evaluations for spot blotch to identify lines with stable spot blotch resistance (Singh et al., 2015). The positions of all the 96 markers consistently associated with spot blotch in this study were compared to previously reported spot blotch associated markers/QTL, whose positions were either available in the RefSeq v1.0 or obtained using their sequences in the nucleotide Basic Local Alignment Search Tool available in Triticeae Toolbox (Blake et al., 2016).

On chromosome 1AL, we observed that significant marker 1A_579403901 was 2.9 Mbps away from spot blotch associated marker S1A_582293281 (Jamil et al., 2018), and might be possibly indicating it or a novel locus. On chromosome 1BL, considering the marker 1B_673569173 that was significant in three panels, we observed that it was: (i) 4.7 Mbps away from marker *hbe248* that was linked to the *Lr46/Sr58/Yr29/Pm39/Ltn2* locus and tagged a minor spot blotch QTL (Lillemo et al., 2013), (ii) in the same position as a spot blotch associated region reported by Gahtyari et al. (2021) between 670.6–673.7 Mbps and (iii) 0.78 Mbps away from marker IWB5678 that was distal to the *Lr46/Sr58/Yr29/Pm39/Ltn2* locus (Singh et al., 1998b; Kolmer et al., 2019). This indicates that marker 1B_673569173 and the other markers in the LD block are closely linked to the *Lr46/Sr58/Yr29/Pm39/Ltn2* locus, thereby providing further evidence to the association of this locus with spot blotch resistance, in addition to partial and durable resistance to rust diseases and powdery mildew (Lillemo et al., 2013). However, we also observed that the markers 1B_673569173 and 1B_673961339 were significant in only three of the seven panels and had average effects of 5.6 and 6.6 on the rAUDPC values, respectively. This is in agreement with Lillemo et al. (2013) who detected the locus in one environment only and designated it as a minor QTL and Singh et al. (1998b) who reported that the *Lr46* gene must be present in combination with other slow rusting genes to impart sufficient resistance in an additive manner. We also observed that the favorable alleles for marker 1B_673569173 were present in 76% of the lines, indicating that a large number of advanced breeding lines from CIMMYT have this spot blotch-associated locus.

On chromosome 2AS, the markers in the region between 718152 and 24002740 bps were in the same position as a previously reported spot blotch associated marker S2A_16824871 (Jamil et al., 2018) and QTL (Gahtyari et al., 2021), besides the 2NS translocation from *Aegilops ventricosa* (Juliana et al., 2019, 2020a,b; Gao et al., 2021). This is a key finding in this study, which is the first report of the association of the 2NS translocation with spot blotch resistance. We have also successfully demonstrated that the spot blotch favorable consensus allele at the 2NS translocation increases grain yield evaluated in the irrigated and moderate-drought stress environment of Obregon, and the irrigated environments of Ludhiana and Pusa. A major implication of this finding is that simultaneous improvement for high grain yield and spot blotch resistance especially in the Indian subcontinent where spot blotch is a serious production constraint can be made by selecting for the 2NS translocation. It is also interesting that the 2NS translocation has been reported to be associated with lodging tolerance, resistance to stripe rust caused by *Puccinia striiformis*, stem rust caused by *Puccinia graminis*, leaf rust caused by *Puccinia triticina*, eyespot caused by *Pseudocercosporella herpotrichoides*, cereal cyst caused by *Heterodera avenae*, root-knot caused by *Meloidogyne* spp. and blast caused by *Magnaporthe oryzae* pathotype *Triticum* (Doussinault et al., 1983; Bariana and Mcintosh, 1993; Jahier et al., 2001; Williamson et al., 2013; Juliana et al., 2018, 2019, 2020a,^2021^; Gao et al., 2021). However, it should be noted that while the markers in the 2NS translocation were significantly associated with spot blotch in only two panels in this study using genome-wide association mapping, the consensus favorable allele at this locus was associated with spot blotch in four of the seven panels and had effects ranging between 4.3 and 6.3 on the spot blotch rAUDPC values. These results indicate that the effect of the 2NS translocation on spot blotch is probably environment-dependent and minor. Nevertheless, the proportion of lines with the spot blotch favorable alleles at the 2NS translocation has increased from 45.2% in panel 2014 to 95.3% in panel 2020, due to the indirect selection for high stripe rust resistance and grain yield (Juliana et al.,b,^2021^).

On chromosome 2AL, the spot blotch associated marker 2A_451012543 was in the same position as the spot blotch QTL, *QSb.bhu-2A* that was located between markers *Xbarc353* (205192882 bps) and *Xgwm445* (682622675 bps) (Kumar et al., 2009) and the favorable allele for this marker was present in a moderate frequency (32.9%) in the breeding lines. On chromosome 3BS, we have reported a 10.1 Mbps region between 627922 and 10737359 bps where several markers were significantly associated with spot blotch in more than one panel and were in the same position as spot blotch associated marker *wPt-1159* (Adhikari et al., 2012) and QTL, *Qcim.3B.1* and *Qcim.3B.2* (Juliana et al., 2019). Considering the positions of the *Sb3* gene-associated markers (Lu et al., 2016) on the Refseq v1.0 (*Xbarc147* – 7104675 bps, *XWGGC3957* – 6233346 bps, and *XWGGC4320* – 5941271 bps), the markers significant in this study are in the same position as the *Sb3* gene. The significant markers that were closest to the *Sb3* gene included 3B_5601689, 3B_6127880, 3B_7237763, 3B_8521721, 3B_8737702 and probably the unaligned markers UN_35335292 and UN_35809999. However, a substantial variation in the favorable allele/consensus favorable allele frequencies at these regions ranging from 16.1 to 86.6% made it elusive to estimate the exact frequency of the *Sb3* gene in the CIMMYT advanced breeding lines. While the high recombination rates at the telomeric ends of the chromosomes lead to rapid breakdown of LD, it is worth highlighting that the spot blotch favorable allele frequency estimate using marker 3B_2280114 was five times higher than marker 3B_627922 that was only 1.6 Mbps away, thereby emphasizing the need for caution when interpreting marker favorable allele frequencies in such regions of the genome.

An interesting finding in this study was the association of grain yield evaluated in Ludhiana, Jabalpur, Pusa, and the irrigated and drought stress environments of Obregon with spot blotch favorable alleles at markers 3B_2280114 and 3B_5601689. While the association of these markers with grain yield and spot blotch was reported in Juliana et al. (2019), we have further extended the analysis to larger datasets and provide strong evidence of the association, implying that selection for favorable alleles at these markers could help obtain higher grain yield and spot blotch resistance. While it is possible that the *Sb3* gene has a favorable pleiotropic effect on grain yield, it is also likely that a closely linked gene is associated with increased grain yield and further studies are needed to provide better insights into this association. However, it should also be mentioned that the markers 3B_2280114 and 3B_5601689 that were associated with a substantial increase in grain yield in Pusa (0.48 and 0.37 t/ha, respectively) had only small effects on the spot blotch rAUDPC values in Agua Fria (highest effects observed were 5.5 and 4.9 on the rAUDPC values, respectively), indicating that they are associated with a minor effect spot blotch locus and further evaluations for both these traits in the same environment are needed.

On chromosome 4AL, significant marker 4A_711331984 was only 2.2 Mbps away from marker BobWhite_c20322_153 that was associated with spot blotch incubation period (Ahirwar et al., 2018) and might be indicating the same locus. On chromosome 5BS, the locus tagged by markers 5B_78260518 and 5B_78260589 that were significant in three panels did not coincide with any previously identified spot blotch associated locus and is indicating a novel spot blotch associated locus. On chromosome 5BL, markers 5B_403782889 and 5B_557138254 significant in this study flanked the *Sb2* gene linked to markers *Xgwm1043* and *Xgwm639* (504301901 bps on the Refseq v1.0) (Kumar et al., 2015). The favorable allele frequency at marker 5B_557138254 which is the significant marker closest to the *Sb2* gene was 25.2% and the highest effect of this marker on the spot blotch rAUDPC value was 5.3, indicating that the effect was minor.

On chromosome 7DS, the significant marker 7D_57903956 was 10.5 Mbps away from the *Sb1* gene and might be linked to the gene. While the percentage of lines with the spot blotch favorable allele at marker 7D_57903956 was 55.4%, the percentage of lines with the consensus favorable allele at the LD block tagged by markers 7D_57903956, 7D_58206696, 7D_58493344, and 7D_58500973 was 64.5%, indicating a moderately high frequency of lines with favorable alleles at this region. We also observed that the maximum effect of the markers at the LD block tagged by markers 7D_57903956, 7D_58206696, 7D_58493344, and 7D_58500973 on spot blotch rAUDPC values was 4.4, indicating that this locus has a minor effect on spot blotch. This observation is in agreement with Lillemo et al. (2013), who also suggested that the effect of the *Sb1* gene on spot blotch is quantitative and it has to be deployed in combination with other resistance genes to confer sufficient levels of resistance.

We have also reported allelic fingerprinting of the spot blotch associated markers, which indicated that although CIMMYT’s advanced breeding lines are quite rich in spot blotch favorable alleles due to extensive breeding efforts (Dubin and Rajaram, 1996), there are opportunities for increasing the proportion of favorable alleles at several markers. One approach that could ideally complement phenotypic selection in increasing the frequencies of favorable alleles for disease resistance is genomic selection, in which dense genome-wide molecular markers are used instead of specific trait-associated markers and selection is done based on the additive effects of multiple loci (Meuwissen et al., 2001; Poland and Rutkoski, 2016; Juliana et al., 2019). Our results also indicated a significant relationship between the percentage of favorable alleles in the lines and their spot blotch, which taken together with the multiple minor effect loci identified to be associated with spot blotch in this study, indicate quantitative genetic control of resistance as also reported previously (Duveiller and Sharma, 2009; Singh et al., 2018). We also observed lower mean rAUDPC values and higher mean percentage of favorable alleles in the HLBSNs compared to the panels in all the cycles, thereby indicating that phenotypic selection for spot blotch resistance was very effective in increasing the mean percentage of favorable alleles in the HLBSNs.

Overall, the genome-wide association mapping and allelic fingerprinting results presented in this study have helped to extend our knowledge on the genetic basis of spot blotch resistance in bread wheat. While we have successfully validated some previously identified spot blotch associated genes/QTL/markers, we have also reported several novel spot blotch associated GBS markers, which could prove very useful for accelerating marker-assisted selection and genomic breeding for spot blotch (Sharma et al., 2007; Duveiller and Sharma, 2009). We have also reported a reference map with the consistent spot blotch associated markers aligned to the reference genome (RefSeq version 1.0) of bread wheat, which will serve as a valuable guide facilitating comparisons with other biparental and association mapping studies for spot blotch. Considering the persistent threat of spot blotch to resource-poor farmers in South Asia, further research and breeding efforts to improve genetic resistance to the disease (Sharma and Duveiller, 2006), identification of novel sources of resistance by screening different germplasms (Kumar et al., 2016a; Bainsla et al., 2020), using high-throughput phenotyping for accurate selection (Kumar et al., 2016b), selecting for and stacking QTL with minor effects (Singh et al., 2018), etc. are essential.

## Data Availability Statement

The datasets presented in this study can be found in online repositories. The names of the repository/repositories and accession number(s) can be found below: https://figshare.com/ and https://doi.org/10.6084/m9.figshare.17195006.v2.

## Author Contributions

PJ performed the analyses and drafted the manuscript. PS, RS, and AJ designed the experiments and supervised the analysis. XH was involved in spot blotch phenotyping. JP and SS were involved in generating the genotyping data. JH-E, VG, LC-H, SM, UK, PB, and MV generated the grain yield phenotyping data. All authors reviewed the article and approved the submitted version.

## Conflict of Interest

The authors declare that the research was conducted in the absence of any commercial or financial relationships that could be construed as a potential conflict of interest.

## Publisher’s Note

All claims expressed in this article are solely those of the authors and do not necessarily represent those of their affiliated organizations, or those of the publisher, the editors and the reviewers. Any product that may be evaluated in this article, or claim that may be made by its manufacturer, is not guaranteed or endorsed by the publisher.

## Footnotes

1
www.jmp.com

2
http://visualization.ritchielab.org/phenograms/plot

## Supplementary Material

Supplementary Figure 1 Manhattan plots showing the marker −log_10_ *p*-values and the chromosomal positions for the marker-days to heading associations in genome-wide association mapping in seven panels. The blue line indicates the threshold of 0.001 to declare the significance of markers and selected markers that were significantly associated with days to heading are indicated.

Supplementary Figure 2 Manhattan plots showing the marker −log_10_ *p*-values and the chromosomal positions for the marker-height associations in genome-wide association mapping in seven panels. The blue line indicates the threshold of 0.001 to declare the significance of markers and selected markers that were significantly associated with height are indicated.

Supplementary Figure 3 Linkage disequilibrium between the markers on chromosome 1B. The standardized disequilibrium coefficients (*D*′) are shown in the lower-left matrix and correlations between alleles at the two marker loci (*r*^2^) are shown in the upper-right matrix.

Supplementary Figure 4 Linkage disequilibrium between the markers on chromosome 2A. The standardized disequilibrium coefficients (*D*′) are shown in the lower-left matrix and correlations between alleles at the two marker loci (*r*^2^) are shown in the upper-right matrix.

Supplementary Figure 5 Linkage disequilibrium between the markers on chromosome 3B. The standardized disequilibrium coefficients (*D*′) are shown in the lower-left matrix and correlations between alleles at the two marker loci (*r*^2^) are shown in the upper-right matrix.

Supplementary Figure 6 Linkage disequilibrium between the markers on chromosome 5B. The standardized disequilibrium coefficients (*D*′) are shown in the lower-left matrix and correlations between alleles at the two marker loci (*r*^2^) are shown in the upper-right matrix.

Supplementary Figure 7 Linkage disequilibrium between the markers on chromosome 7D. The standardized disequilibrium coefficients (*D*′) are shown in the lower-left matrix and correlations between alleles at the two marker loci (*r*^2^) are shown in the upper-right matrix.

Supplementary Figure 8 Linkage disequilibrium between the unaligned markers. The standardized disequilibrium coefficients (*D*′) are shown in the lower-left matrix and correlations between alleles at the two marker loci (*r*^2^) are shown in the upper-right matrix.

Supplementary Figure 9 Boxplots with the spot blotch relative area under the disease progress curve (rAUDPC) values in different panels, for lines with favorable alleles (alleles that have a decreasing effect on spot blotch) and non-favorable alleles (alleles that have an increasing effect on spot blotch) at spot blotch associated markers. The boxplots are only shown for the panels where the mean differences in rAUDPC values for the favorable and non-favorable alleles were significant.

Supplementary Table 1 Markers significantly associated with spot blotch in panels 2014, 2015, 2016, 2017, 2018, 2019, and 2020 after Bonferroni correction for multiple testing at an α level of 0.2 along with markers that were consistently associated with spot blotch in at least two panels at a *p*-value threshold of 0.001. The markers that were not significant after Bonferroni correction for multiple testing are indicated along with the number of panels in which the markers were significant.

Supplementary Table 2 Allelic fingerprinting of markers that were consistently associated with spot blotch in more than one panel (1 = favorable allele that has a decreasing effect on the spot blotch relative area under the disease progress curve values, 0 = non-favorable allele that has an increasing effect on the spot blotch relative area under the disease progress curve values, 0.5 = heterozygote).

Supplementary Table 3 Lines with 67–83 spot blotch favorable alleles at 96 markers that were consistently associated with spot blotch in more than one panel (1 = favorable allele that has a decreasing effect on the spot blotch relative area under the disease progress curve values, 0 = non-favorable allele that has an increasing effect on the spot blotch relative area under the disease progress curve values, 0.5 = heterozygote).
