# Supplementary figures and images for: Genome-Wide Association Mapping Indicates Quantitative Genetic Control of Spot Blotch Resistance in Bread Wheat and the Favorable Effects of Some Spot Blotch Loci on Grain Yield
Source: Front Plant Sci. 2022 Mar 3;13:835095. doi: 10.3389/fpls.2022.835095 (PMC8928540; doi:10.3389/fpls.2022.835095)

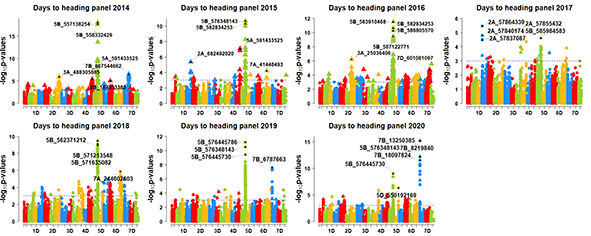

Supplement: Supplementary Figure 1 — Manhattan plots showing the marker −log10 p-values and the chromosomal positions for the marker-days to heading associations in genome-wide association mapping in seven panels. The blue line indicates the threshold of 0.001 to declare the significance of markers and selected markers that were significantly associated with days to heading are indicated. [file Image_1.TIFF]

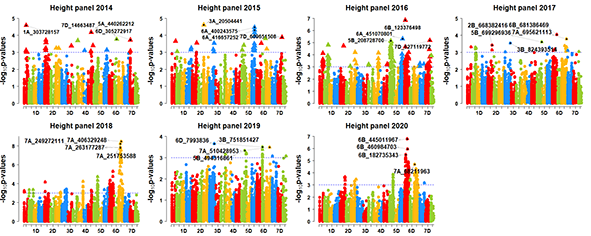

Supplement: Supplementary Figure 2 — Manhattan plots showing the marker −log10 p-values and the chromosomal positions for the marker-height associations in genome-wide association mapping in seven panels. The blue line indicates the threshold of 0.001 to declare the significance of markers and selected markers that were significantly associated with height are indicated. [file Image_2.TIFF]

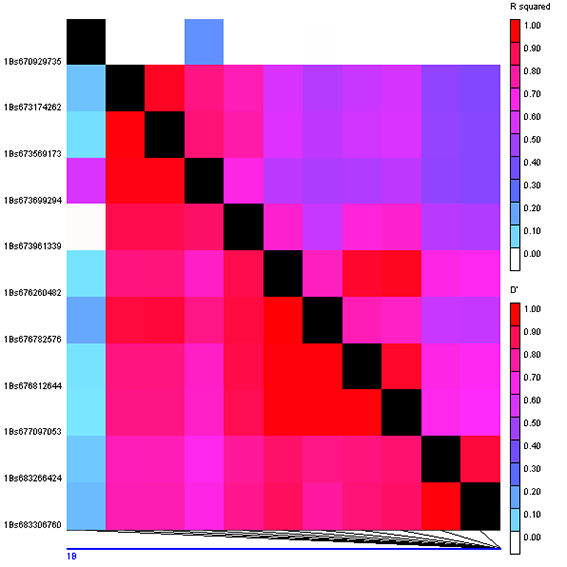

Supplement: Supplementary Figure 3 — Linkage disequilibrium between the markers on chromosome 1B. The standardized disequilibrium coefficients (D′) are shown in the lower-left matrix and correlations between alleles at the two marker loci (r2) are shown in the upper-right matrix. [file Image_3.TIFF]

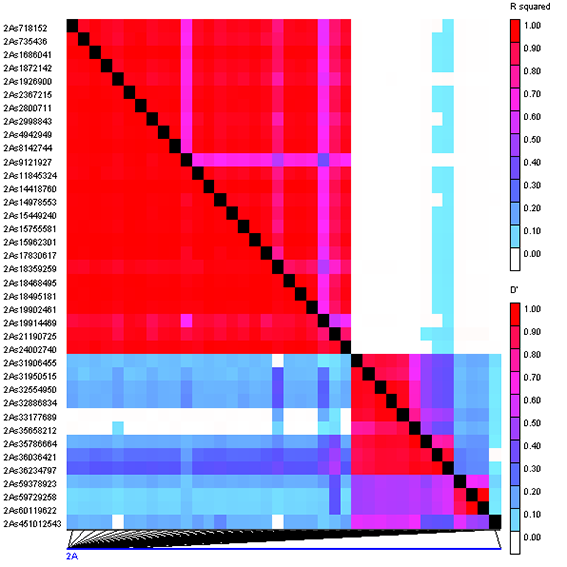

Supplement: Supplementary Figure 4 — Linkage disequilibrium between the markers on chromosome 2A. The standardized disequilibrium coefficients (D′) are shown in the lower-left matrix and correlations between alleles at the two marker loci (r2) are shown in the upper-right matrix. [file Image_4.TIFF]

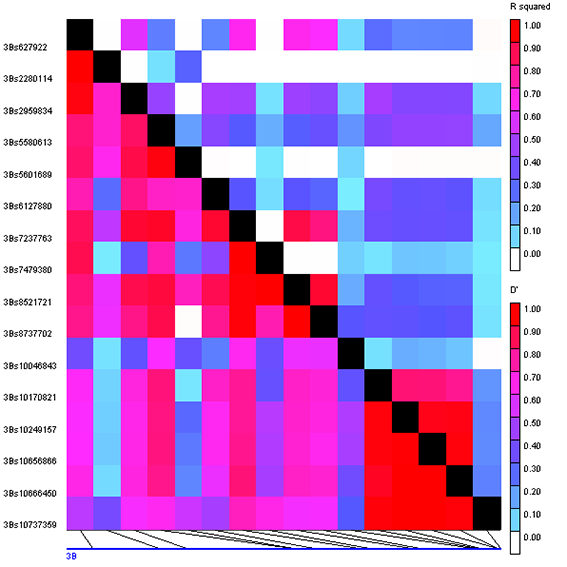

Supplement: Supplementary Figure 5 — Linkage disequilibrium between the markers on chromosome 3B. The standardized disequilibrium coefficients (D′) are shown in the lower-left matrix and correlations between alleles at the two marker loci (r2) are shown in the upper-right matrix. [file Image_5.TIFF]

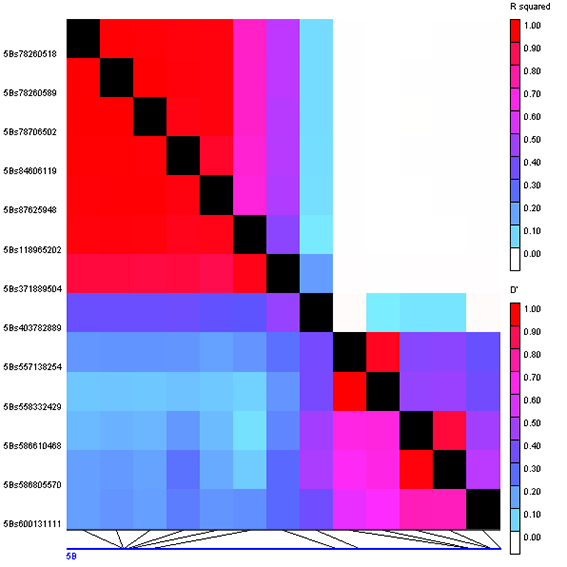

Supplement: Supplementary Figure 6 — Linkage disequilibrium between the markers on chromosome 5B. The standardized disequilibrium coefficients (D′) are shown in the lower-left matrix and correlations between alleles at the two marker loci (r2) are shown in the upper-right matrix. [file Image_6.TIFF]

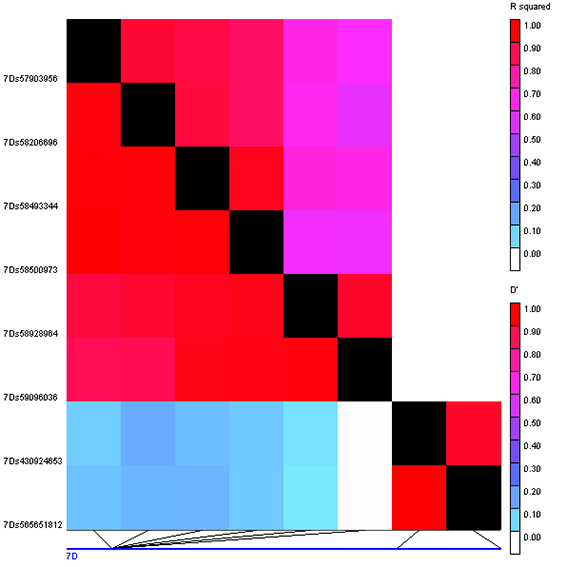

Supplement: Supplementary Figure 7 — Linkage disequilibrium between the markers on chromosome 7D. The standardized disequilibrium coefficients (D′) are shown in the lower-left matrix and correlations between alleles at the two marker loci (r2) are shown in the upper-right matrix. [file Image_7.TIFF]

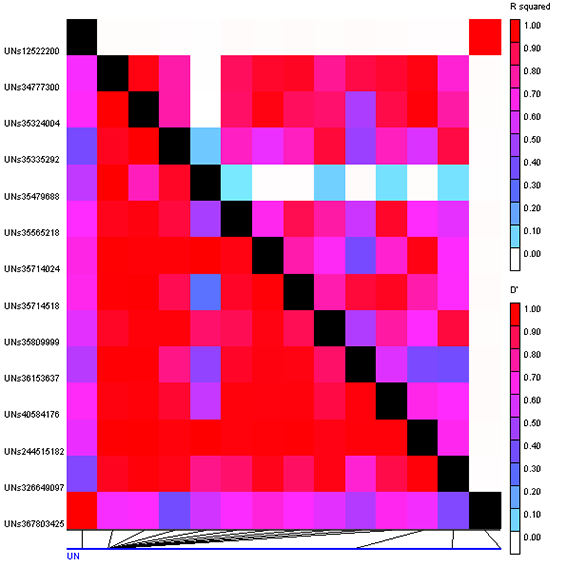

Supplement: Supplementary Figure 8 — Linkage disequilibrium between the unaligned markers. The standardized disequilibrium coefficients (D′) are shown in the lower-left matrix and correlations between alleles at the two marker loci (r2) are shown in the upper-right matrix. [file Image_8.TIFF]

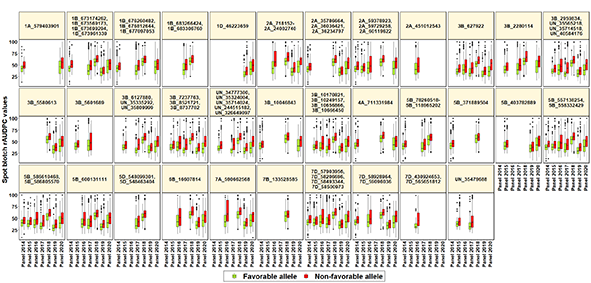

Supplement: Supplementary Figure 9 — Boxplots with the spot blotch relative area under the disease progress curve (rAUDPC) values in different panels, for lines with favorable alleles (alleles that have a decreasing effect on spot blotch) and non-favorable alleles (alleles that have an increasing effect on spot blotch) at spot blotch associated markers. The boxplots are only shown for the panels where the mean differences in rAUDPC values for the favorable and non-favorable alleles were significant. [file Image_9.TIFF]
